# I smell something weird - Ictal olfactory hallucinations in patients with primary brain tumors

**DOI:** 10.1192/j.eurpsy.2024.659

**Published:** 2024-08-27

**Authors:** M. Mousinho, D. Antão, A. Soares, T. Pimentel, D. Salgado, M. Fernandes

**Affiliations:** ^1^Mental Health Department, Local Health Unit of Baixo Alentejo, Beja; ^2^Neurology Department; ^3^Neurophysiology Laboratory, Portuguese Institute of Oncology, Lisboa, Portugal

## Abstract

**Introduction:**

Ictal olfactory hallucinations (the experience of a smell due to a focal seizure in the absence of an environmental stimulus for the sensation) are rare. They often appear in a context of a brain tumor located in the orbitofrontal or mesotemporal region. However, their accurate prevalence, etiology and anatomical origin remains unclear, as few studies focused on this type of seizures specifically.

**Objectives:**

To evaluate the clinical, neurophysiological and imaging characteristics of patients with brain tumors and olfactory seizures.

**Methods:**

We present a 3-year retrospective patient record study carried out at the Portuguese Institute of Oncology in Lisbon. Clinical records of 572 patients admitted due to a primary Central Nervous System (CNS) tumor, for their first neuro-oncology appointment, between July 2020 and July 2023, were reviewed.

**Results:**

8 patients with olfactory seizures were identified. Five were men. The mean age was 57.75 (ages between 15 and 70 years old). In seven patients, olfactory seizures constituted the initial clinical presentation of the tumor. In two patients, focal olfactory seizures had progression to bilateral tonic clonic. Most seizures were perceived as unpleasant (smells of metal, ammonia, “hot blood”, “dead bodies” were described). Tumors involved the temporal lobe in all patients, the insula in two of them and, for the majority, the lesion was right-sided. Six patients were diagnosed with Glioblastoma IDH wildtype (Grade 4, WHO), one patient with Oligodendroglioma, IDH-mutated and 1p/19q-codeleted (Grade 2, WHO) and the pediatric patient with a diffuse pediatric type high-grade glioma, H3 and IDH wildtype. The average follow-up time was 6.8 months, two patients died.

**Conclusions:**

This is the first retrospective study carried out in Portugal that documents the prevalence of olfactory seizures in patients with primary CNS tumors. Given the scarce literary evidence, we consider that olfactory seizures may be more frequent than documented, particularly in the presentation of brain tumors. As so, active semiological investigation may contribute to an earlier diagnosis.

**Disclosure of Interest:**

None Declared

